# Supplementary figures and images for: Analysis of the Applicability of microRNAs in Peripheral Blood Leukocytes as Biomarkers of Sensitivity and Exposure to Fractionated Radiotherapy towards Breast Cancer
Source: Int J Mol Sci. 2021 Aug 13;22(16):8705. doi: 10.3390/ijms22168705 (PMC8395710; doi:10.3390/ijms22168705)

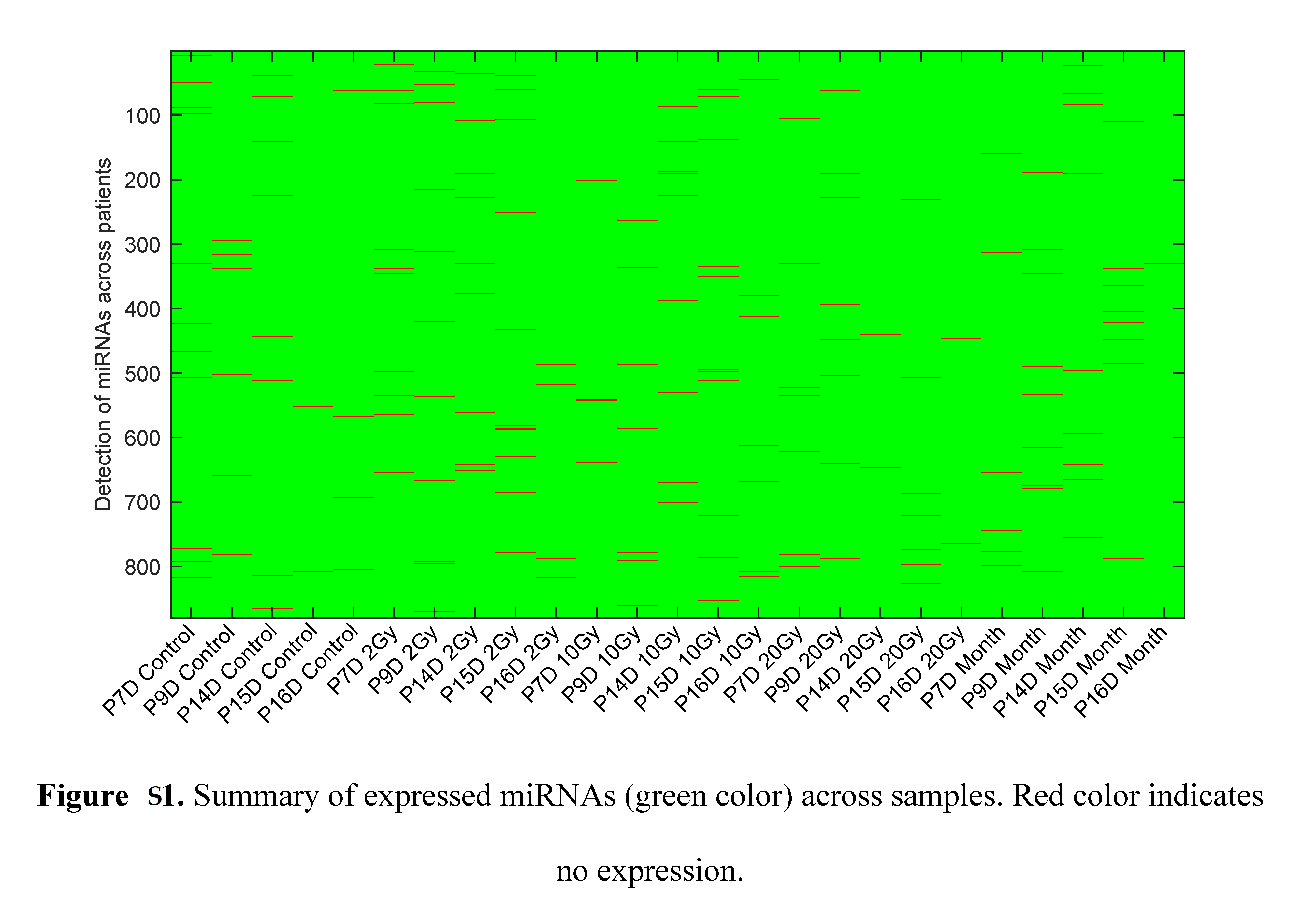

Supplement: Supplementary file 1 [file ijms-22-08705-s001.zip › Figure S1.tiff]

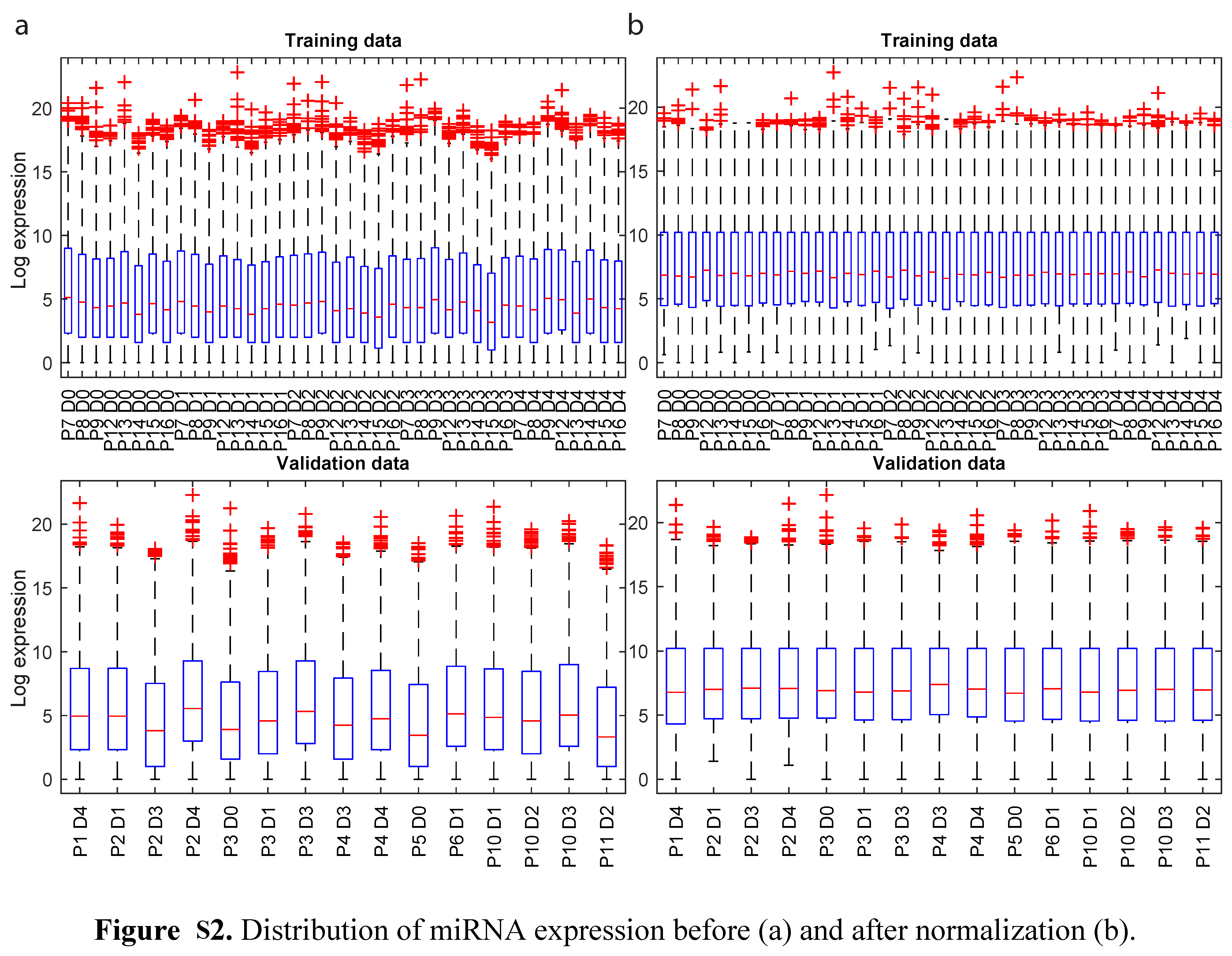

Supplement: Supplementary file 1 [file ijms-22-08705-s001.zip › Figure S2.tiff]

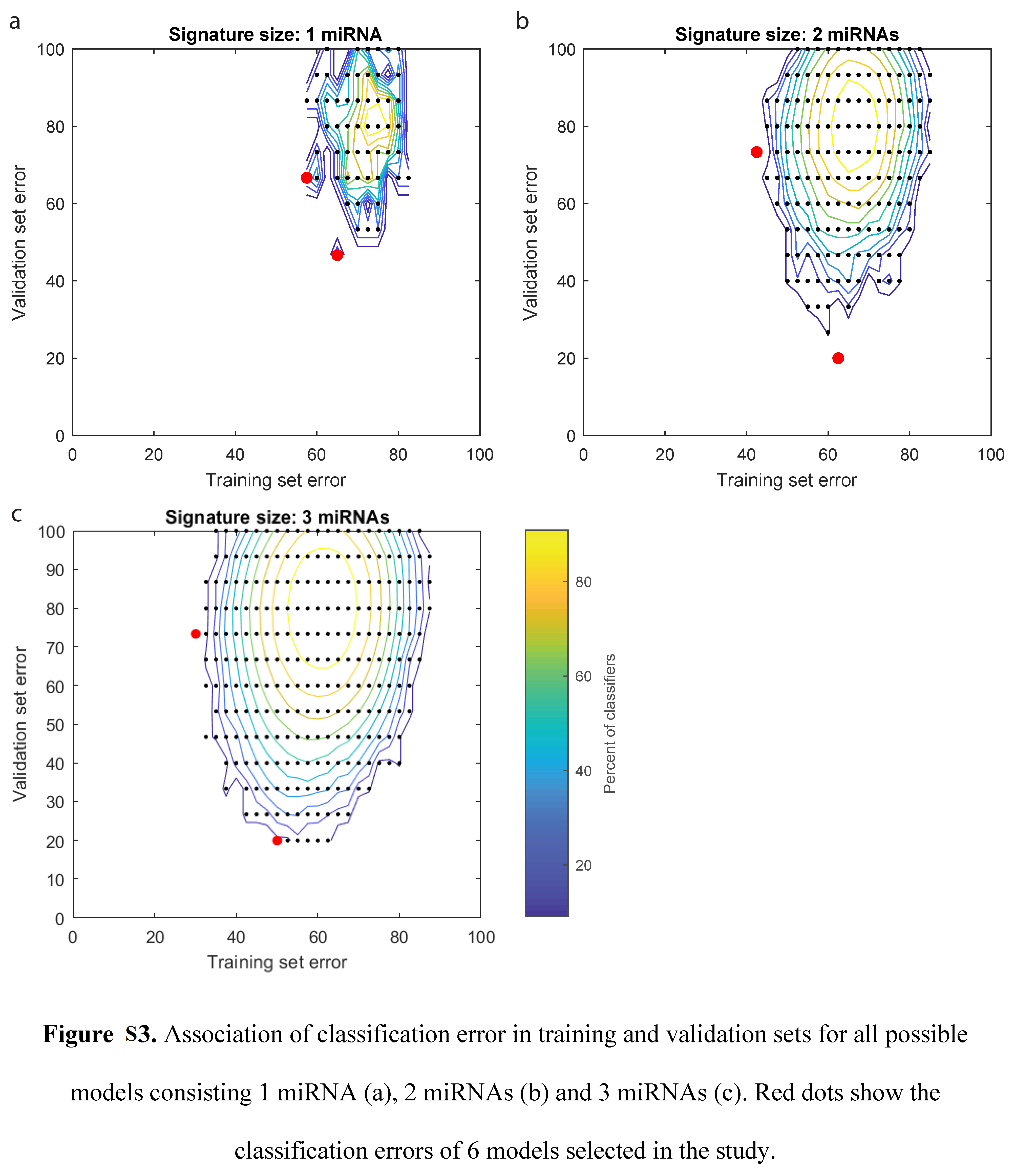

Supplement: Supplementary file 1 [file ijms-22-08705-s001.zip › Figure S3.tiff]
